# Changes in Lignin and Polysaccharide Components in 13 Cultivars of Rice Straw following Dilute Acid Pretreatment as Studied by Solution-State 2D ^1^H-^13^C NMR

**DOI:** 10.1371/journal.pone.0128417

**Published:** 2015-06-17

**Authors:** Hiroshi Teramura, Kengo Sasaki, Tomoko Oshima, Shimpei Aikawa, Fumio Matsuda, Mami Okamoto, Tomokazu Shirai, Hideo Kawaguchi, Chiaki Ogino, Masanori Yamasaki, Jun Kikuchi, Akihiko Kondo

**Affiliations:** 1 Department of Chemical Science and Engineering, Graduate School of Engineering, Kobe University, Rokkodaicho, Nada-ku, Kobe, Hyogo, Japan; 2 Organization of Advanced Science and Technology, Kobe University, Rokkodaicho, Nada-ku, Kobe, Hyogo, Japan; 3 Department of Bioinformatic Engineering, Graduate School of Information Science and Technology, Osaka University, Yamadaoka, Suita, Osaka, Japan; 4 RIKEN Biomass Engineering Program, Suehiro-cho, Tsurumi-ku, Yokohama, Kanagawa, Japan; 5 Food Resources Education and Research Center, Graduate School of Agricultural Science, Kobe University, Uzurano, Kasai, Hyogo, Japan; 6 RIKEN Center for Sustainable Resource Science, Suehiro-cho, Tsurumi-ku, Yokohama, Kanagawa, Japan; 7 Graduate School of Medical Life Science, Yokohama City University, Suehirocho, Tsurumi-ku, Yokohama, Japan; 8 Graduate School of Bioagricultural Sciences and School of Agricultural Sciences, Nagoya University, Furo-cho, Chikusa-ku, Nagoya, Japan; National Research Council of Italy, ITALY

## Abstract

A renewable raw material, rice straw is pretreated for biorefinery usage. Solution-state two-dimensional (2D) ^1^H-^13^ C hetero-nuclear single quantum coherence (HSQC) nuclear magnetic resonance (NMR) spectroscopy, was used to analyze 13 cultivars of rice straw before and after dilute acid pretreatment, to characterize general changes in the lignin and polysaccharide components. Intensities of most (15 of 16) peaks related to lignin aromatic regions, such as *p*-coumarate, guaiacyl, syringyl, *p*-hydroxyphenyl, and cinnamyl alcohol, and methoxyl, increased or remained unchanged after pretreatment. In contrast, intensities of most (11 of 13) peaks related to lignin aliphatic linkages or ferulate decreased. Decreased heterogeneity in the intensities of three peaks related to cellulose components in acid-insoluble residues resulted in similar glucose yield (0.45–0.59 g/g-dry biomass). Starch-derived components showed positive correlations (*r* = 0.71 to 0.96) with glucose, 5-hydroxymethylfurfural (5-HMF), and formate concentrations in the liquid hydrolysates, and negative correlations (*r* = –0.95 to –0.97) with xylose concentration and acid-insoluble residue yield. These results showed the fate of lignin and polysaccharide components by pretreatment, suggesting that lignin aromatic regions and cellulose components were retained in the acid insoluble residues and starch-derived components were transformed into glucose, 5-HMF, and formate in the liquid hydrolysate.

## Introduction

In order for ethanol to be classified as an environmentally friendly fuel, it must be produced from abundant and renewable resources, such as lignocellulosic materials [1]. One of the largest biomass feedstocks is lignocellulosic waste from agricultural residues such as straw, particularly rice (*Oryza sativa*) straw [2]. Forage rice cultivars are also promising candidates for the production of aboveground biomass. Rice straw is mainly composed of cellulose, hemicellulose, and lignin [3]. Due to its complex chemical structure, bioethanol production from rice straw requires pretreatment to facilitate subsequent enzymatic hydrolysis [4].

Dilute acid pretreatment has been studied widely due to its low cost, convenience, and effectiveness for a broad range of lignocellulosic biomass sources [5–8]. Dilute acid pretreatment produces an acid-insoluble residue and liquid hydrolysate. The acid-insoluble residue is composed primarily of cellulose and lignin [9]. This cellulose is a major source of glucose following enzymatic hydrolysis. The composition and structure of the acid-insoluble residue affects the efficiency of enzymatic hydrolysis [10–12]. However, understanding of how dilute acid pretreatment alters the lignin and polysaccharide components and acid-insoluble residue in various kinds of rice straws remains limited [13].

Solution-state two-dimensional (2D) ^1^H-^13^C hetero-nuclear single quantum coherence nuclear magnetic resonance (HSQC-NMR) spectroscopy is an effective high-resolution tool for rapid and reproducible fingerprinting of numerous polysaccharide and lignin components in environmental sources [14–17]. A recent study reported the development of a simple and rapid method using ball-milled plant biomass and dimethyl sulfoxide (DMSO)-d_6_/pyridine-d_5_ organic solvent that enables analysis of whole plant cell wall samples. In this method, the NMR peak intensity semi-quantitatively reflects the amount of biomass components [18–20]. Several NMR studies have shown changes in lignocellulose during plant growth [21] and compositional differences among various plant tissues [22–23]. Therefore, 2D ^1^H-^13^C HSQC-NMR spectroscopy is useful for investigating in detail the lignin and polysaccharide components of rice straw before and after dilute acid pretreatment.

The liquid hydrolysate contains sugars used for microbial fermentation, such as glucose and xylose, and also contains fermentation inhibitors, such as 5-hydroxymethylfurfural (5-HMF) and formate [24]. Previous research showed a positive correlation between the glucose concentration in the liquid hydrolysate and the starch content of various types of rice straw [25]. 2D ^1^H-^13^C HSQC-NMR spectroscopy can elucidate the relationship between the lignin and polysaccharide components in rice straw and the concentrations of various components of the liquid hydrolysate (e.g., glucose, xylose, 5-HMF, and formate) and the relationship between the components in rice straw and the amount (weight) of acid-insoluble residue.

The primary aim of the present study was to characterize changes in lignin and polysaccharide components resulting from dilute acid pretreatment. To achieve this aim, 2D ^1^H-^13^C HSQC-NMR spectroscopy was used to examine samples of 13 cultivars of rice straw before and after dilute acid pretreatment (i.e., raw biomass and acid-insoluble residues). These results from multiple numbers of rice cultivars could reveal the common effects of dilute acid pretreatment on lignin and polysaccharide components. A secondary aim was to analyze the relationships between 2D ^1^H-^13^C HSQC-NMR data and the concentrations of glucose, xylose, 5-HMF, and formate in the liquid hydrolysate and acid-insoluble residue yield. The results of these relationships revealed that starch in the rice straw was one of important factors to determine the amount of these compounds in the liquid hydrolysate.

## Materials and Methods

### Plant materials

The 13 rice (*Oryza sativa* L.) straw cultivars examined in the study were grown in 2011 in an experimental field located at the Food Resources Education and Research Center, Kobe University (Kasai City, Hyogo Prefecture, Japan) using a single procedure. Whole plants were harvested 45 to 50 days after flowering and allowed to dry in the field for 3 days. The grains were removed and the straw was powdered using a blender (WB-1; TGK, Tokyo, Japan) fitted with a 2-mm screen.

### Sugar analysis

For sugar analysis, each sample (1.5 μL) was mixed with 1.5 μL of 0.1% (w/w) Ribitol, and the mixture was then dried in a vacuum concentrator (7810010; Labconco, Kansas City, MO, USA). The dried residue was dissolved in 100 μL of 20 mg/mL methoxyamine hydrochloride in pyridine and incubated at 30°C for 90 min, after which 50 μL of N-methyl-N-trimethylsilyltrifluoroacetamide was added and the sample was incubated at 37°C for 30 min. A 10-μL aliquot of the sample solution was subjected to gas chromatography-mass spectrometry (GC-MS) on a GC-MS-2010 plus system (Shimadzu, Kyoto, Japan) under the following conditions: column, Agilent CP-Sil 8CB-MS (30 m × 0.25 mm); carrier gas, helium; injection temperature, 230°C; oven temperature, 80°C at *t* = 0 to 2 min, then to 330°C at 15°C/min.

### By-product analysis

Acetone (900 μL) was added to 100 μL of liquid hydrolysate and mixed thoroughly, then the sample was centrifuged at 21,880 × *g* at room temperature for 10 min. An aliquot (10 μL) of the resulting supernatant was subjected to GC-MS analysis on a GC-MS-2010 plus instrument (Shimadzu). The following conditions were used for analysis of 5-HMF and furfural: column, Agilent CP-Sil 8CB-MS (30 m × 0.25 mm); carrier gas, helium; injection temperature, 250°C; oven temperature, 50°C at *t* = 0 to 5 min, then to 280°C at 20°C/min. Formate was analyzed under the following conditions: column, Agilent DB-FFAP (60 m × 0.25 mm); carrier gas, helium; injection temperature, 250°C; oven temperature, 100°C at *t* = 0 to 5 min, then to 230°C at 10°C/min.

### Dilute acid pretreatment

Dilute acid pretreatment was performed using an HHE-19G-U laboratory-scale thermostirrer (Koike Precision Instruments, Kanagawa, Japan). The reactor had a total volume of 100 mL and was equipped with an electric heater and was capable of magnetic agitation. The optimal temperature and agitation speed for pretreatment were determined according to a previous report [26]. Rice straw powder (6 g) was suspended in 80 mL of 1% (v/v) sulfuric acid and incubated at 180°C for 45 min with agitation at 200 rpm. Following pretreatment, the liquid hydrolysate and acid-insoluble residue were separated by filtration. The acid-insoluble residue was washed with deionized water, neutralized to pH 7.0, then dried. The weight of the residue was determined using an XS105DU electronic balance (Mettler Toledo, Greifensee, Switzerland). The liquid hydrolysate was neutralized to pH 5.0 by the addition of calcium hydroxide powder. The sugar and fermentation inhibitor concentrations in the liquid hydrolysate were determined by GC-MS using the conditions described in section 2.2 above.

### Enzymatic saccharification

Enzymatic saccharification of the acid-insoluble residue (10% dry weight) was performed by adding 0.3 M citrate buffer (pH 4.8) and cellulase (Cellic CTec2, Novozyme, Bagsvaerd, Denmark) to the residue at a concentration of 66 filter paper unit (FPU)/g dry biomass. Tetracycline (40 μg/mL) and cycloheximide (30 μg/mL) were added to prevent the growth of any potential microbial contaminants. The reaction mixture was incubated at 50°C for 72 h in a PPS-2000 ChemiStation (Tokyo Rikakikai, Tokyo, Japan) with agitation at 120 rpm. Enzymatic saccharification was stopped by rapid chilling on ice, followed by centrifugation at 21,880 × *g* for 10 min at 4°C. The sugars in the supernatant were analyzed as described in section 2.2 above.

### NMR spectroscopy

Solubilized lignocelluloses were prepared similarly to previous studies [20]. Briefly, dried cell-wall material was further ground in a Pulverisette 5 ball mill (Fritsch GmbH, Idar-Oberstein, Germany) to yield 300 mg of stock powder of raw rice straw and the acid-insoluble residue. The ball-milled powder (30 mg) was mixed with 600 μL of DMSO-d_6_:pyridine-d_5_ (4:1), heated at 50°C for 30 min in a Thermomixer Comfort (Eppendorf AG, Hamburg, Germany), then centrifuged at 20,380 × *g* for 5 min. The supernatant was transferred to 5-mm ϕ NMR tubes and subjected to NMR analysis. NMR spectra were recorded on an AvanceIII HD-600 instrument (Bruker, Billerica, MA, USA) equipped with a 5-mm TXI-cryo probe operated at 600 MHz for ^1^H and 125 MHz for ^13^C analyses. The temperature of all NMR samples was maintained at 313 K. Chemical shifts were referenced to the methyl group of DMSO-d_6_ at ^13^C = 40.03 ppm and ^1^H = 2.582 ppm. Two-dimensional ^1^H-^13^C HSQC-NMR spectra were collected using echo/antiecho gradient selection (the “hsqcetgp” pulse program in the Bruker library). Forty-eight regions of interest (ROI) were identified based on previously assigned chemical shifts [20] Details of the ROI for raw rice straw biomass are described in Table 1 and S1 Fig. Peak intensity of each component was calculated using tetramethylsilane (TMS) as internal standard.

Relative peak intensity was calculated using following formula with same amount of raw rice straw (before pretreatment) and the acid-insoluble residue (after pretreatment).

$$\text{Relative peak intensity}=\frac{Peak\;intensity\;after\;pretreatment}{Peak\;intensity\;before\;pretreatment}$$

**Table 1:** Annotation of 2D ^1^H-^13^C HSQC-NMR spectral peaks.

ROI No.	^1^H-chemical shift (ppm)	^13^C-chemical shift (ppm)		Annotation	
			Large category		Small category
ROI.1	7.68	130.45	Lignin	Lignin. aromatic	*p*-Coumarate
ROI.2	7.64	130.35		Lignin. aromatic	*p*-Coumarate
ROI.3	7.57	130.32		Lignin. aromatic	*p*-Coumarate
ROI.4	7.51	130.34		Lignin. aromatic	*p*-Coumarate
ROI.5	7.00	116.17		Lignin. aromatic	*p*-Coumarate
ROI.6	6.70	114.54		Lignin. aromatic	*p*-Coumarate
ROI.7	7.13	116.15		Lignin. aromatic	Guaiacyl
ROI.8	7.05	115.14		Lignin. aromatic	Guaiacyl
ROI.9	6.92	114.93		Lignin. aromatic	Guaiacyl
ROI.10	6.82	115.00		Lignin. aromatic	Guaiacyl
ROI.11	6.98	104.49		Lignin. aromatic	Syringyl
ROI.12	6.89	104.45		Lignin. aromatic	Syringyl
ROI.13	7.38	128.26		Lignin. aromatic	*p*-Hydroxyphenyl
ROI.14	4.31	61.87		Lignin. aromatic	Cinnamyl alcohol end group
ROI.15	7.27	123.58		Lignin. aromatic	Ferulate
ROI.16	7.49	111.61		Lignin. aromatic	Ferulate
ROI.17	3.96	56.12		Others	Methoxyl
ROI.18	3.88	55.98		Others	Methoxyl
ROI.19	5.18	83.17		Lignin. aliphatic	5-5/4-O-β
ROI.20	3.67	87.71		Lignin. aliphatic	5-5/4-O-β
ROI.21	5.19	72.98		Lignin. aliphatic	β-O-4
ROI.22	5.15	72.23		Lignin. aliphatic	β-O-4
ROI.23	5.07	72.27		Lignin. aliphatic	β-O-4
ROI.24	3.88	60.72		Lignin. aliphatic	β-O-4-H/G
ROI.25	3.50	60.81		Lignin. aliphatic	β-O-4-H/G
ROI.26	5.01	71.25		Lignin. aliphatic	β-O-4-H/G
ROI.27	5.01	72.05		Lignin. aliphatic	β-O-4-H/G
ROI.28	4.29	86.70		Lignin. aliphatic	β-O-4-S
ROI.29	3.85	52.17		Lignin. aliphatic	β-5
ROI.30	4.53	97.97	Hemicellulose	Saccharide	β-D-Xylopyranoside
ROI.31	5.12	92.78		Saccharide	α-D-Xylopyranoside
ROI.32	3.50	63.54		Saccharide	β-D-Xylopyranoside
ROI.33	3.42	63.58		Saccharide	β-D-Xylopyranoside
ROI.34	4.19	63.43		Saccharide	β-D-Xylopyranoside
ROI.35	4.11	63.69		Saccharide	β-D-Xylopyranoside
ROI.36	4.73	99.70		Saccharide	2-O-Ac-Xylopyranoside
ROI.37	4.79	73.85		Saccharide	2-O-Ac-Xylopyranoside
ROI.38	4.68	101.86		Saccharide	2-O-Ac-Xylopyranoside
ROI.39	5.10	75.26		Saccharide	3-O-Ac-Xylopyranoside
ROI.40	5.25	72.87		Saccharide	3-O-Ac-Mannopyranoside
ROI.41	4.52	102.12		Saccharide	β-D-Xylopyranoside & α-D-Glucopyranoside
ROI.42	5.70	107.77		Saccharide	α-L-Arabinofuranoside
ROI.43	4.62	97.18	Cellulose	Saccharide	β-D-Glucopyranoside
ROI.44	5.20	92.65		Saccharide	α-D-Glucopyranoside
ROI.45	4.61	103.19		Saccharide	(1→4)-β-D-Glucopyranoside & (1→3)-β-D-Glucopyranoside
ROI.46	5.37	100.62	Others	Saccharide	α-L- Fructopyranoside
ROI.47	5.29	101.10		Saccharide	α-L- Fructopyranoside

## Results and Discussion

### 2D ^1^H-^13^C HSQC-NMR analysis of changes in biomass components following dilute acid pretreatment

**Fig 1:**
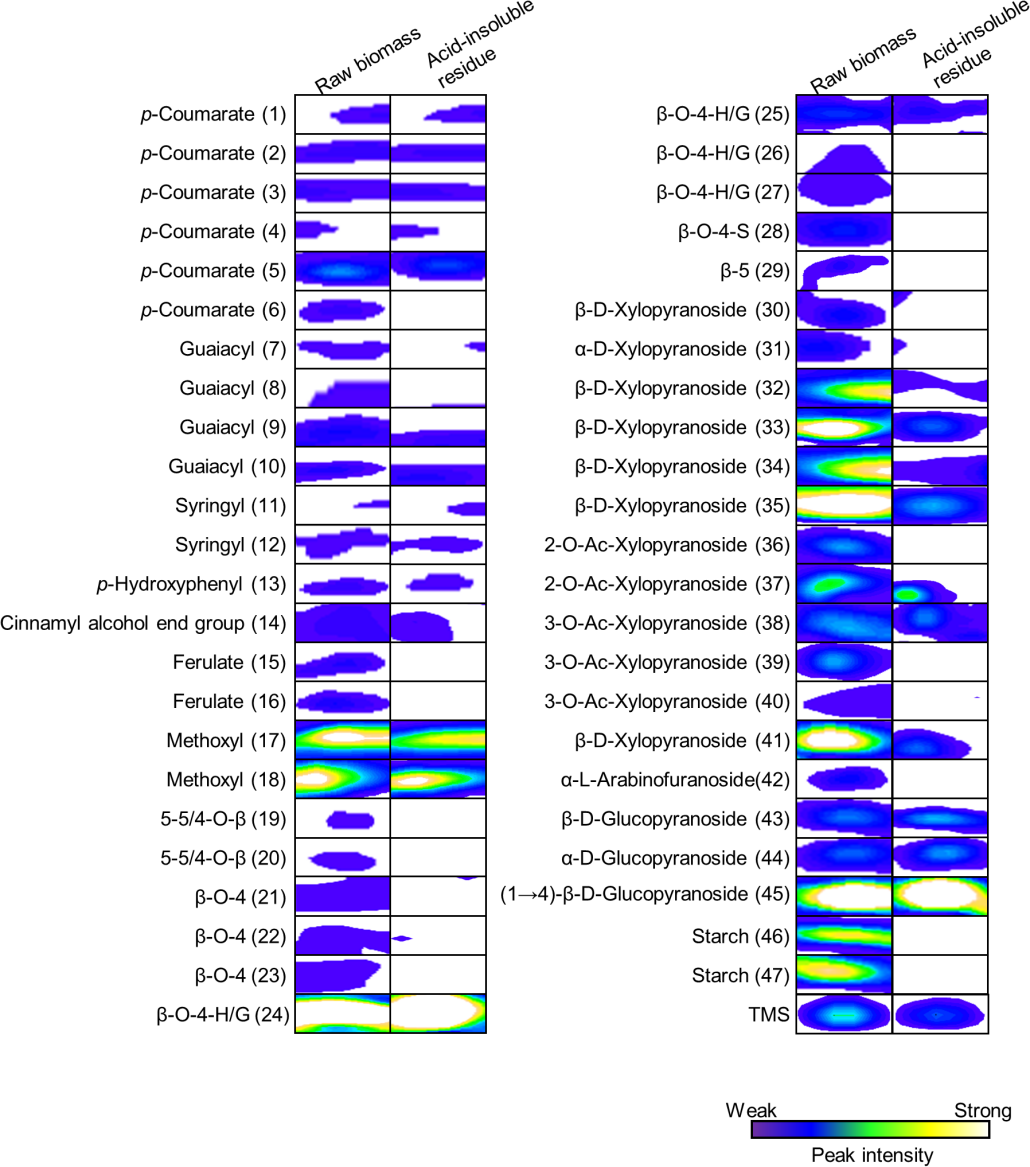
Comparison of ROI of NMR peaks of raw rice straw, Nipponbare, and the corresponding acid-insoluble residue. Left portion of figure shows NMR peaks of raw rice straw. Right portion of figure shows NMR peaks of acid-insoluble residue after dilute acid pretreatment of Nipponbare rice straw. NMR analysis was done using same amount of raw rice straw and the acid-insoluble residue.

**Fig 2:**
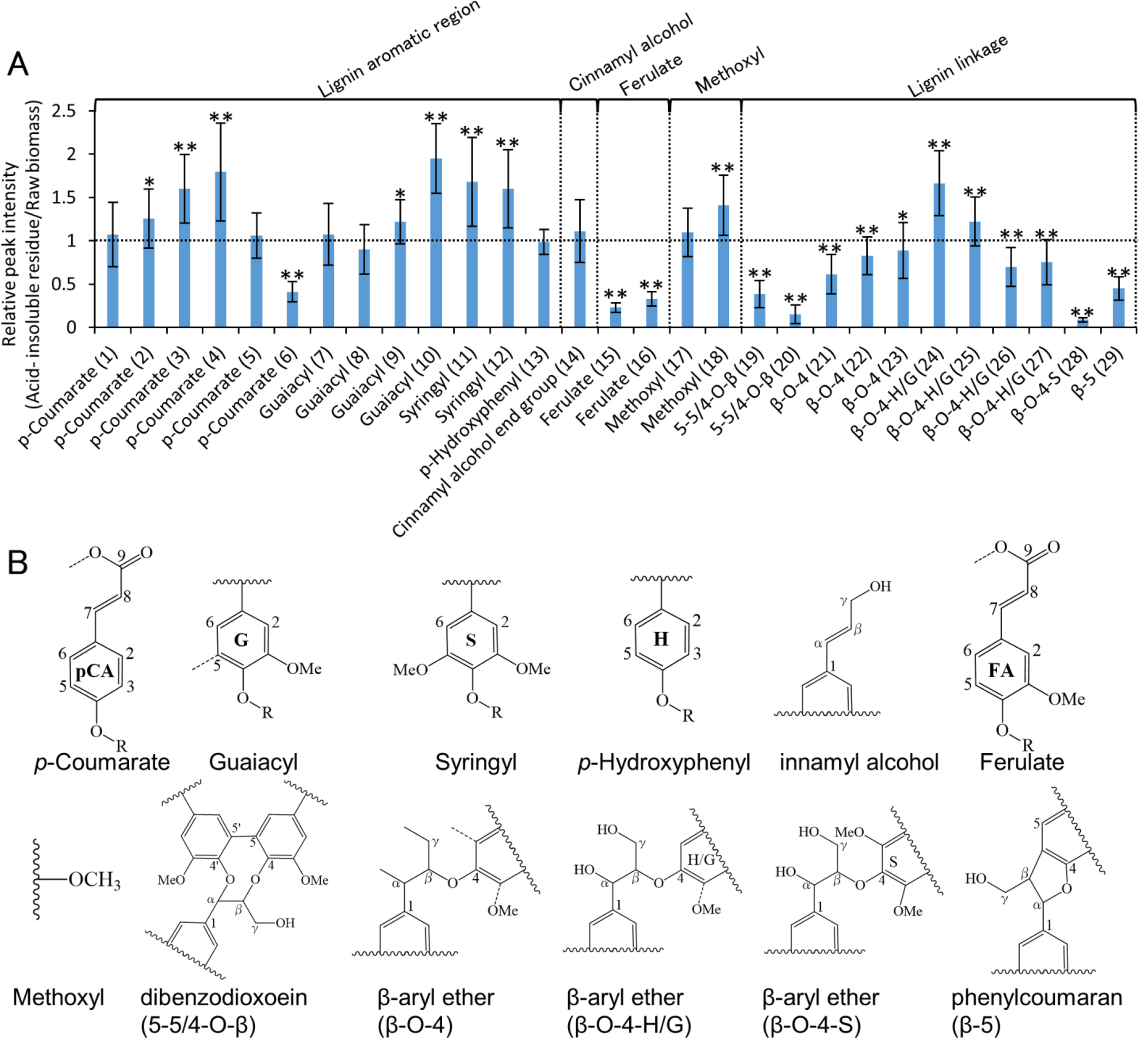
Relative peak intensity of acid-insoluble residue to that of raw biomass of 13 cultivars of rice straw. (A) Relative peak intensity of lignin with same amount of raw rice straw and the acid-insoluble residue. Vertical axes indicate relative peak intensity (acid-insoluble residue/raw biomass). Peak intensities of the acid-insoluble residue were significantly different than those of raw biomass (*: *P*<0.05; **: *P*<0.01). ROI numbers from Table 1 are shown in parentheses. (B) Structural formula of each compounds.

**Fig 3:**
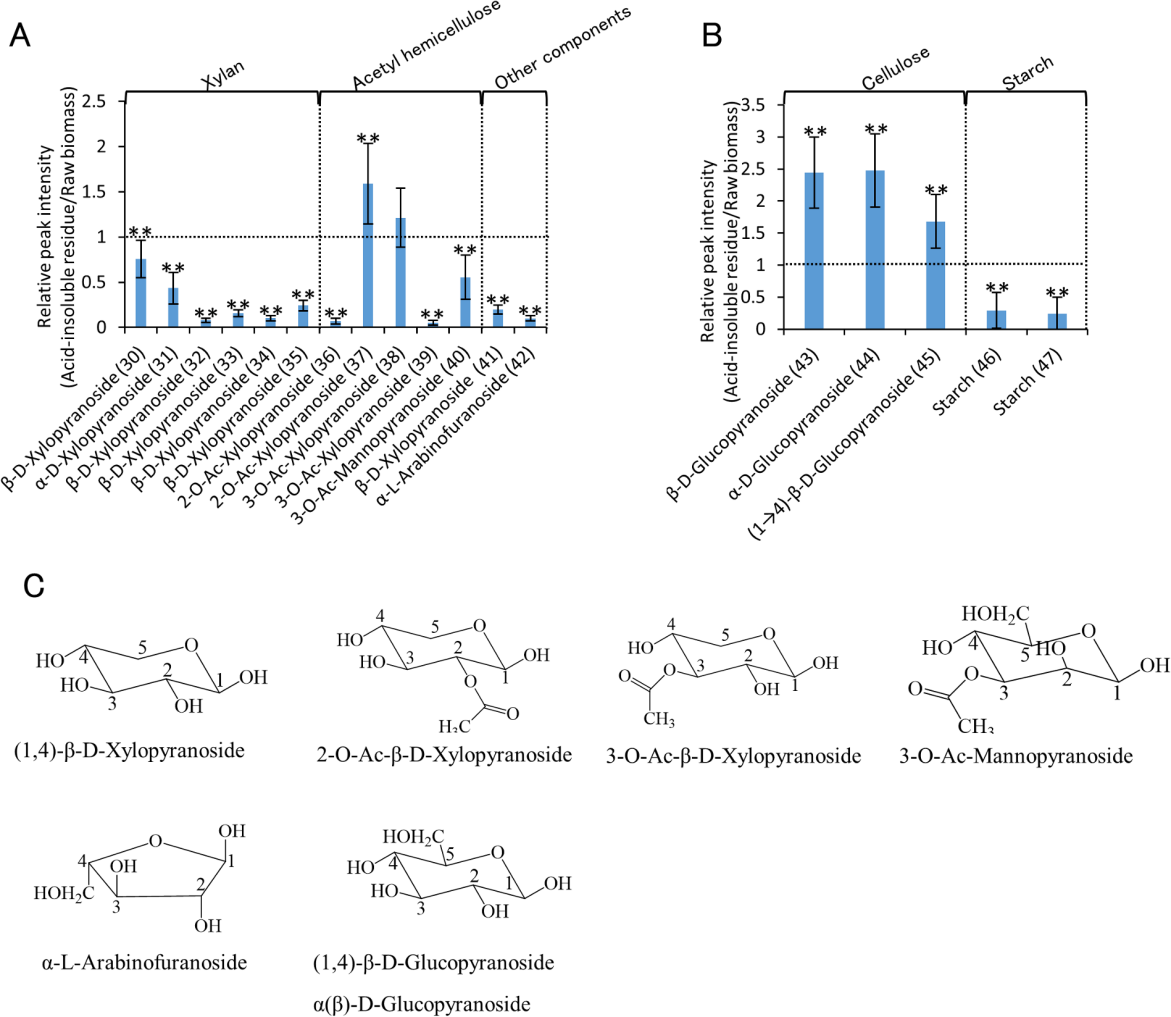
Relative peak intensity of acid-insoluble residue to that of raw biomass of 13 cultivars of rice straw. Relative peak intensity of (A) hemicellulose and (B) cellulose and starch with same amount of raw rice straw and the acid-insoluble residue. Vertical axes indicate relative peak intensity (acid-insoluble residue/raw biomass). Peak intensities of the acid-insoluble residue were significantly different than those of raw biomass (*: *P*<0.05; **: *P*<0.01). ROI numbers from Table 1 are shown in parentheses. B) Structural formula of each compounds.

A comparison of the 2D ^1^H-^13^C HSQC-NMR spectral peak intensities of raw rice straw and acid-insoluble residue revealed changes in the lignin and polysaccharide components following dilute acid pretreatment (for analysis of Nipponbare in Fig 1). The positions and annotations of ROI are listed in Table 1. To determine the general trend of changes in the components, the relative peak intensities of raw biomass and acid-insoluble residue produced by dilute acid pretreatment were calculated for the 13 cultivars examined in this study (Fig 2, Fig 3 and S2 Fig).

#### Lignin

Lignin-related peaks showed variable tendencies. Most signals related to aromatic regions, such as *p*-coumarate, guaiacyl, syringyl, and *p*-hydroxyphenyl (ROI 1–13 except ROI 6), and that of the cinnamyl alcohol end group (ROI 14), increased or were unchanged after dilute acid pretreatment (Fig 2). Similarly, signals related to methoxyl (ROI 17 and 18), a functional moiety of lignin, either increased or remained unchanged after dilute acid pretreatment. In contrast, signals related to ferulate (ROI 15 and 16), which bridges lignin and polysaccharides [27–29] decreased. A decrease in the number of ferulate is favorable for enzymatic hydrolysis because close proximity between the lignin and polysaccharide moieties prevents physical access by enzymes [30–31]. Moreover, most signals related to aliphatic linkages, such as 5-5/4-O-β (dibenzodioxin: ROI 19 and 20), β-O-4 (β-aryl ether: ROI 21, 22, and 23), β-O-4-H/G (β-aryl ether p-hydroxyphenyl/guaiacyl: ROI 26 and 27), β-O-4-S (β-aryl ether syringyl: ROI 28), and β-5 (phenylcoumaran: ROI 29), decreased. Similarly, a previous study showed a 36% decrease in β-O-4 linkages in switchgrass after dilute acid pretreatment [32]. The remaining signals related to β-O-4-H/G (ROI 24 and 25), increased. These results suggest that most aromatic regions are retained in the acid-insoluble residue, whereas most lignin aliphatic linkages and ferulate between the lignin and polysaccharides are decreased after dilute acid pretreatment.

#### Hemicellulose

Most hemicellulose-related signals (ROI 30–36, 39–42) decreased after dilute acid pretreatment. Only two xylopyranoside-related signals (ROI 37 and 38) increased or remained unchanged. These results correlated with previous research showing that hemicellulose is removed during pretreatment [7]. The decrease in the arabinofuranoside-related signal (ROI 42) correlated with the decrease in ferulate-related signals (ROI 15 and 16) in lignin because ferulate cross-bridges arabinoxylan chains and lignin in grasses [27–29].

#### Cellulose

Cellulose-related signals (ROI 43–45) increased after dilute acid pretreatment. This result correlated with previous research showing that cellulose is more recalcitrant than hemicellulose [7].

#### Other signals

Two other signals (ROI 46 and 47) decreased following dilute acid pretreatment. These two related signals are reportedly derived from starch in potatoes, chicory, and corn [12].

A previous study reported that dilute acid pretreatment of rice straw enhances the efficiency of enzymatic hydrolysis [4]. Remarkably, our analyses of 13 cultivars of rice straw showed that most lignin aromatic regions are retained in the acid-insoluble residue. In contrast, lignin aliphatic linkages, ferulate bridges, and the amount of hemicellulose decrease. The aliphatic linkage, β-O-4, is the most abundant linkage in lignin [33]. The ether bond in the β-O-4 linkage is readily cleaved by formic acid [34]. As observed with formic acid, dilute acid pretreatment cleaved the β-O-4 linkage. The resulting depolymerization of lignin would significantly affect the enzymatic hydrolysis efficiency.

### Decrease in the heterogeneity of components in the acid-insoluble residues from 13 cultivars of rice straw

**Fig 4:**
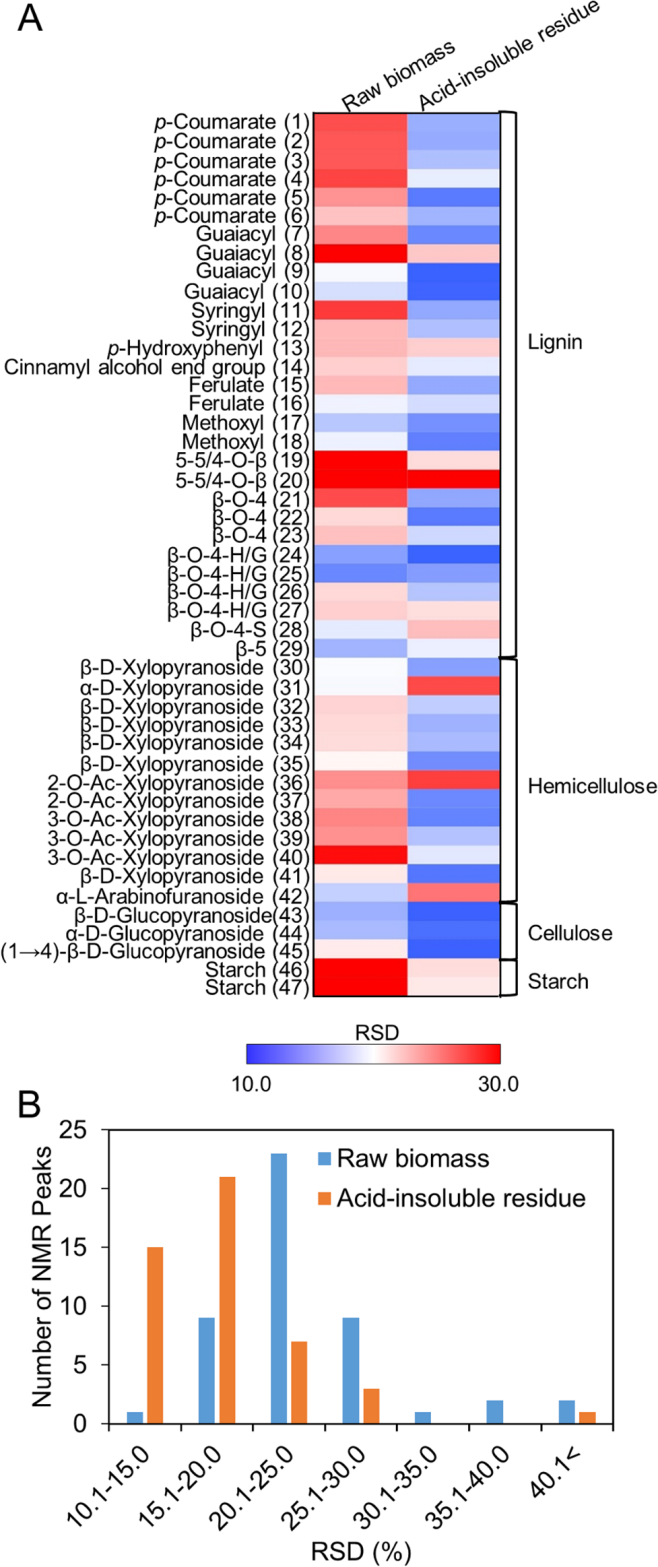
Variations in the lignin and polysaccharide components in the 13 cultivars of rice straw. (A) Relative standard deviation (RSD) of 2D ^1^H-^13^C HSQC-NMR peak intensities of raw biomass and the corresponding acid-insoluble residue (after dilute acid pretreatment) for the 13 cultivars of rice straw. ROI for which the RSD was <10.0 are indicated in blue and those for which the RSD was >2.0 are indicated in red. Fold-change increase or decrease is indicated by the shade of red or blue according to the scale bar. (B) Distribution of RSD values for raw biomass (blue) and acid-insoluble residue (red).

The lignin and polysaccharide components in the acid-insoluble residue affect the efficiency of enzymatic hydrolysis [8–10]. Therefore, the relative standard deviation (RSD) of the intensity of each peak among the 13 cultivars of rice straw was calculated (Fig 4A), and the RSD distribution is shown in Fig 4B. Interestingly, 37 peaks (77.1% of the total peaks) in the acid-insoluble residue showed an RSD of ≤20% (15 peaks: 10.1–15.0%; 22 peaks: 15.1–20.0%). However, only 10 peaks (20.8% of total peaks) showed an RSD of ≤20.0% in untreated rice straw. Of the 15 peaks showing an RSD of ≤15% in the acid-insoluble residues, 3 peaks (ROI 43–45) were annotated as cellulose components and would contribute to a decrease in the heterogeneity of the acid-insoluble residue due to substantial increases in their levels after dilute acid pretreatment. Eight peaks (ROI 5, 7, 9, 10, 17, 18, 22, and 24) were annotated as lignin components. Another 3 peaks (ROI 35, 39, and 41) annotated as hemicellulose components decreased in intensity after pretreatment due to liberation into the liquid hydrolysate. Finally, the intensity of one peak, ROI 38, annotated as hemicellulose, was unchanged by pretreatment. The above results indicate that variations of biomass components among 13 cultivars were decrease following dilute acid pretreatment.

To elucidate the effect of the components on glucose productivity, we analyzed the yield of glucose from the acid-insoluble residues of the 13 cultivars (right column in Table 2). The decrease in the heterogeneity of the components, particularly cellulose, in the acid-insoluble residues following dilute acid pretreatment resulted in similar glucose yields (0.45–0.59 g/g-biomass). In contrast, Studer et al. showed that the lignin content in samples of hot water–pretreated *Populous trichocarpa* residues varies, resulting in different glucose yields upon enzymatic hydrolysis [35]. Collectively, these results show that dilute acid pretreatment of rice straw, regardless of cultivar, produces an acid-insoluble residue that provides similar yields of glucose, and thus, rice straw pretreated in this manner can be used as a substrate for bioethanol production.

**Table 2:** Concentrations of liquid hydrolysate constituents, acid-insoluble residue yield, and glucose yield from acid-insoluble residue that were obtained from raw biomass (75 g/L).

Cultivar	Glucose (g/L)	Xylose (g/L)	Acid insoluble residue yield (%)	Furfural (mM)	5-HMF (mM)	Formate (mM)	Glucose yield from acid insoluble residue (g/ g biomass)
Hoshiaoba	10.1±0.1	6.5±0.5	45.6±0.2	7.3±1.3	2.4±0.4	27.0±1.8	0.48±0.02
Kusanohoshi	5.5±0.1	7.9±0.4	51.6±1.4	8.5±0.9	1.8±0.1	16.2±2.6	0.45±0.01
Leafstar	11.6±0.3	6.2±0.4	43.9±1.0	5.0±1.6	2.7±0.6	28.5±1.8	0.50±0.02
Makimizuho	8.4±0.4	7.4±0.6	49.3±0.6	7.5±1.6	2.2±0.4	21.9±3.3	0.46±0.01
Mizuhochikara	4.2±0.1	8.8±0.7	49.8±0.8	7.6±0.9	1.0±0.1	16.8±0.6	0.48±0.01
Mogumoguaoba	8.0±0.1	7.6±0.2	49.4±1.1	7.1±0.8	2.1±0.1	30.6±1.2	0.47±0.02
Momiroman	3.9±0.2	8.4±0.1	55.0±0.9	6.8±1.0	1.0±0.0	15.7±0.2	0.55±0.00
Nipponbare	11.2±0.5	6.2±0.4	43.6±0.6	5.2±0.5	2.6±0.3	27.7±0.8	0.50±0.02
Nishiaoba	7.2±0.2	7.8±0.4	52.6±1.8	7.0±0.6	1.9±0.2	25.2±2.1	0.54±0.01
Ruriaoba	6.3±0.2	8.5±0.3	51.0±1.4	6.4±1.1	2.1±0.1	28.4±2.1	0.59±0.01
Tachiaoba	8.2±0.2	7.6±0.7	55.7±0.5	6.7±1.1	1.9±0.1	28.2±2.1	0.49±0.01
Tachisugata	5.3±0.3	8.0±0.0	52.7±0.5	5.6±1.3	2.0±0.3	19.0±2.3	0.53±0.01
Tachisuzuka	13.1±0.6	5.9±0.4	41.2±0.4	6.1±0.7	3.1±0.3	42.3±1.9	0.46±0.01

### Positive correlations between glucose, 5-HMF, and formate concentrations in the liquid hydrolysate and starch-derived components

**Fig 5:**
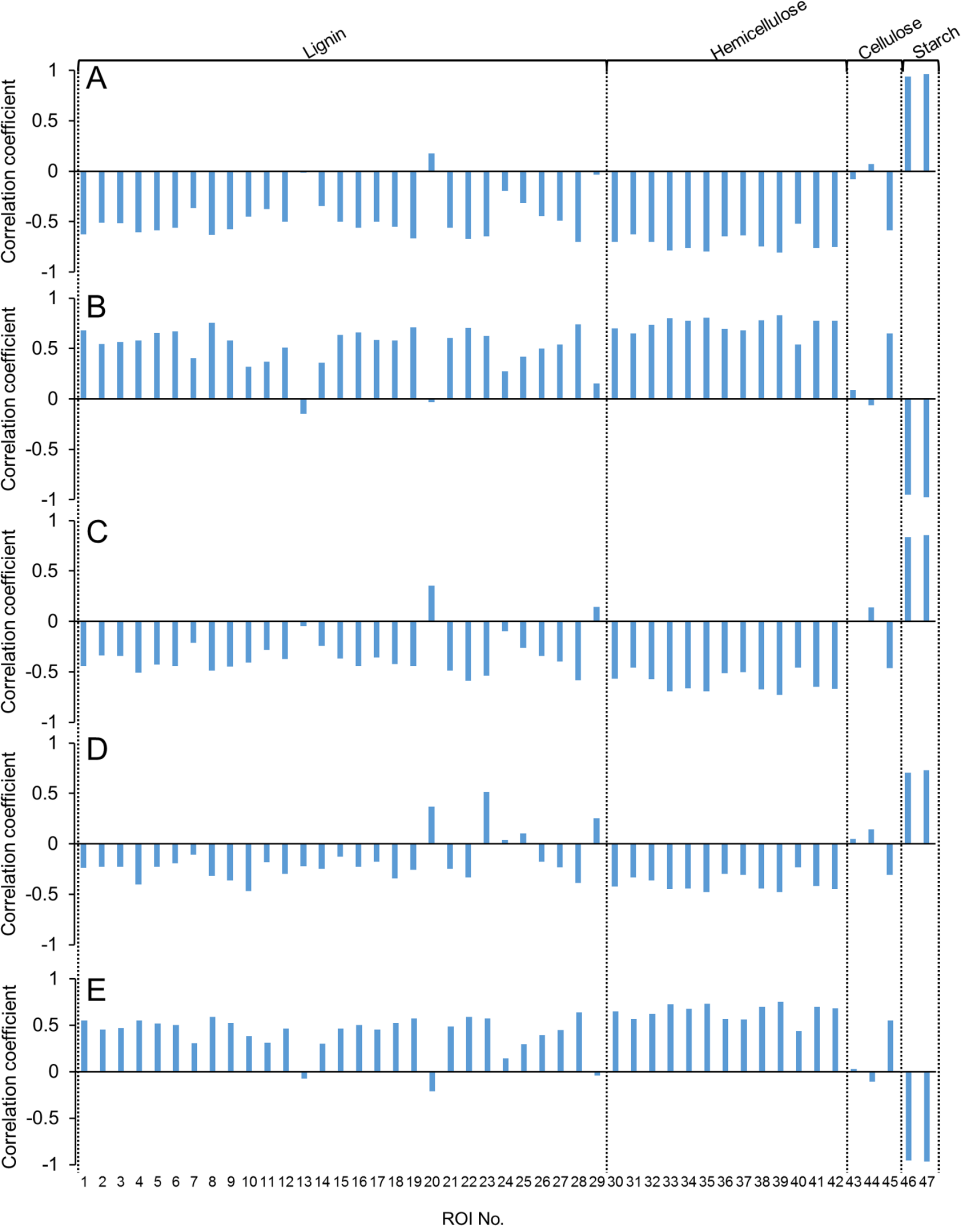
Relationship between NMR peak intensities of raw biomass and the concentrations of glucose (A), xylose (B), 5-HMF (C), and formate (D) in liquid hydrolysate and the acid-insoluble residue yield (E). Correlation coefficients are indicated by the vertical axes. Plus and minus values indicate a positive or negative relationship, respectively.

A previous study reported that the starch content of rice straw correlates with the glucose concentration of the liquid hydrolysate [22]. Therefore, it is important to determine the underlying relationships between lignin and polysaccharide components and the various products resulting from dilute acid pretreatment. To elucidate these relationships, the correlation between each 2D ^1^H-^13^C HSQC-NMR peak and the composition of the liquid hydrolysate or the acid-insoluble residue yield produced was determined for each of the 13 cultivars of rice straw (Fig 5). NMR spectral peaks could be classified into two groups ([ROI 46 and 47] and [ROI 33–35, 38, 39, and 42]) showing opposing tendencies (S1 Table). Starch-related NMR spectra peaks (ROI 46 and 47) were positively correlated (r = 0.71 to 0.96) with the concentrations of glucose, 5-HMF, and formate in liquid hydrolysate and negatively correlated (r = –0.95 to—0.97) with xylose concentration in the liquid hydrolysate and the acid-insoluble residue yield. In contrast, hemicellulose-related NMR spectra peaks (ROI 33–35, 38, 39, 41, and 42) were negatively correlated (r = –0.42 to—0.81) with the concentrations of glucose, 5-HMF, and formate in the liquid hydrolysate but positively correlated (r = 0.68 to 0.82) with xylose concentration in the liquid hydrolysate and the acid-insoluble residue yield.

**Fig 6:**
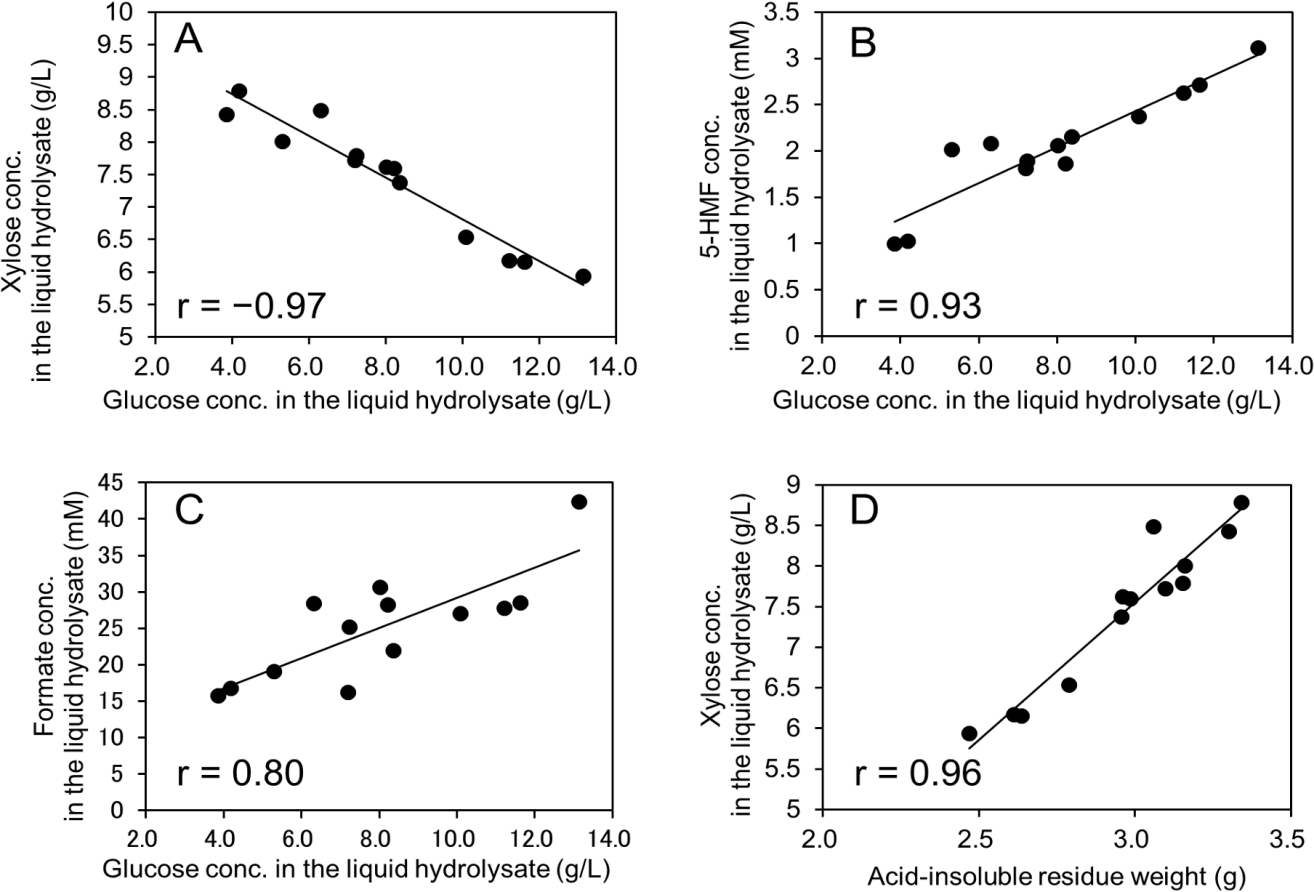
Relationship between the concentrations of glucose, 5-HMF, and formate in the liquid hydrolysate and the acid-insoluble residue yield. (A) Negative relationship between glucose and xylose concentrations. (B) Positive relationship between glucose and 5-HMF concentrations. (C) Positive relationship between glucose and formate concentrations. (D) Positive relationship between xylose concentration and the acid-insoluble residue yield.

**Fig 7:**
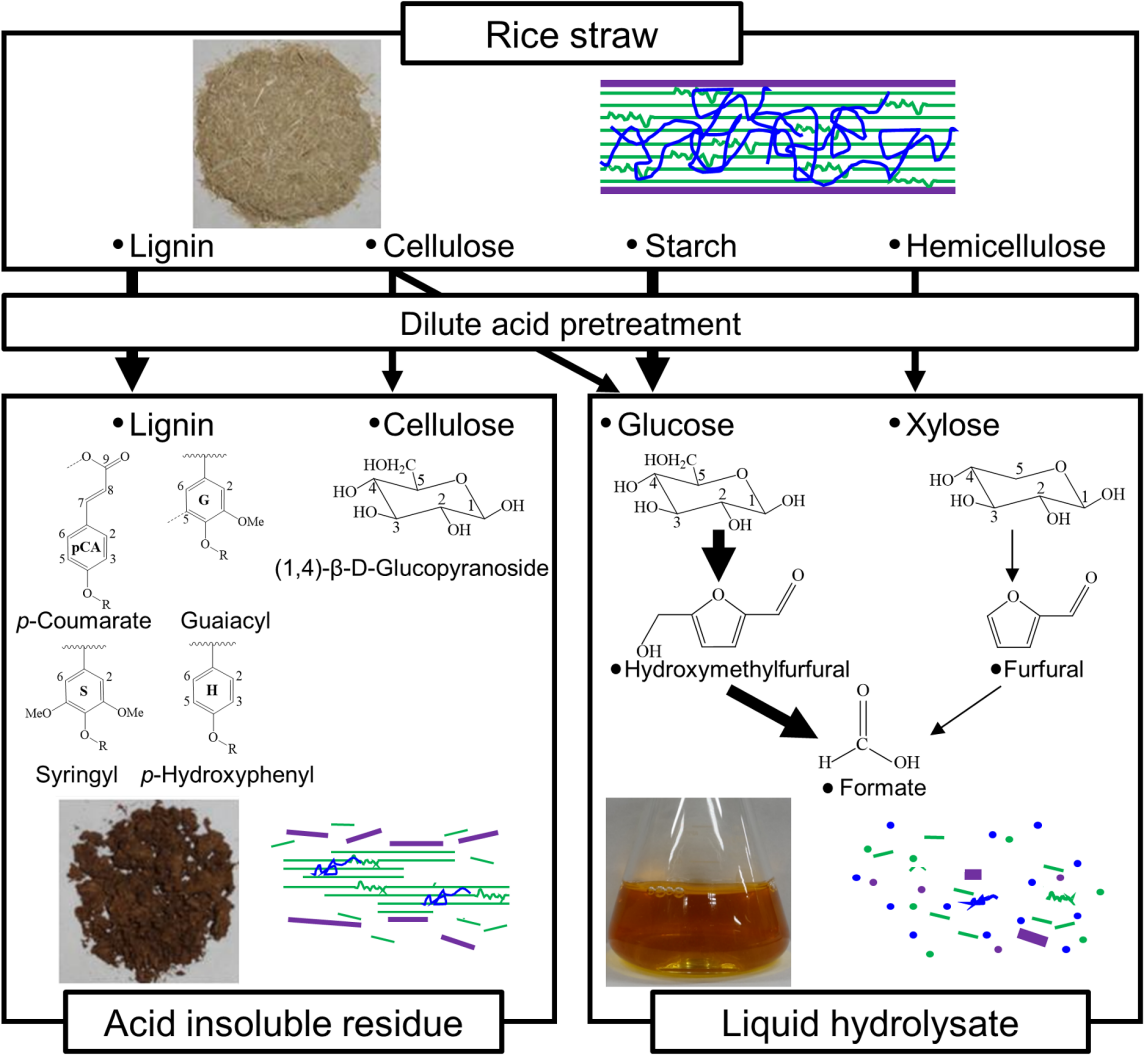
Fate of lignin and polysaccharides components in rice straws following dilute acid pretreatment. Lignin aromatic regions were retained in the acid insoluble residues. Starch-derived components positively affected glucose, 5-HMF, and formate concentrations in the liquid hydrolysate.

To further investigate the above-mentioned relationships observed in 2D ^1^H-^13^C HSQC-NMR analyses, the relationships between the glucose concentration and the concentrations of xylose, 5-HMF, and formate in the liquid hydrolysate and the relationship between the acid-insoluble residue yield and xylose concentration were investigated (Fig 6). As expected, the glucose concentration was positively correlated with the concentrations of 5-HMF and formate (r = 0.93 and 0.80, respectively) as 5-HMF is derived from hexoses and formate is derived from 5-HMF (and furfural) (Fig 6B and 6C) [36]. Interestingly, the glucose concentration negatively correlated with the xylose concentration (r = –0.97) in liquid hydrolysate (Fig 6A). This results suggested that fractionation between glucose and xylose is occurring in soluble sugars following dilute acid pretreatment (Fig 7). Also, the positive relationship (r = 0.96) between xylose concentration in the hydrolysate and the acid-insoluble residue yield (which is composed mainly of cellulose and lignin) (Fig 6D) was interesting because xylan in hemicellulose is the main source of xylose in the liquid hydrolysate. This results suggested that fractionation between glucose in soluble sugars and insoluble sugars following dilute acid pretreatment is also occurring, assuming that lignin content was constant (Fig 7).

The results of the present study showed that the starch content in rice straw serve as good indicators of the xylose, formate, and 5-HMF concentrations in the liquid hydrolysate and of the acid-insoluble residue yield generated by dilute acid pretreatment. Teramura et al. reported that xylose in the liquid hydrolysate and the acid-insoluble residue are generated from the cell wall components, hemicellulose (liberating xylose) and cellulose (found in the acid-insoluble residue), respectively [25]. Another study reported that the levels of non-structural carbohydrates such as starch and soluble sugars are negatively correlated with cell-wall components [37]. Therefore, the concentration of glucose in the liquid hydrolysate is positively correlated with that of starch but negatively correlated with the concentrations of cell wall components, xylose in the liquid hydrolysate, and the acid-insoluble residue (Fig 7). Thereby, it was suggested that 5-HMF and formate in the liquid hydrolysate were derived from starch in rice straws.

## Conclusions

NMR data showed that dilute acid pretreatment leads to changes in the lignin and polysaccharide components in 13 cultivars of rice straw. Pretreatment facilitates the removal of lignin aliphatic linkages and ferulate rather than lignin aromatic regions (Fig 7). A decrease in the heterogeneity of the acid-insoluble residue might affect to similar glucose yields after cellulase hydrolysis. NMR results also indicated that components derived from starch positively affected the glucose, 5-HMF and formate amounts in the liquid hydrolysate resulting from pretreatment. 2D ^1^H-^13^C HSQC NMR analysis revealed common tendencies in the fate of lignin and polysaccharides and that starch would be important source of glucose and above fermentation inhibitors in the liquid hydrolysate during dilute acid pretreatment. These observations would contribute to the screening of rice cultivars those are suitable for downstream bioethanol production.

## Supporting Information

S1 Fig Position of ROI. (A) 2D ^1^H-^13^C HSQC NMR spectrum of the raw biomass (Nipponbare). Squares I, II, III, and IV in (A) correspond to (B), (C), (D), and (E). Gray squares show the position of the ROI. Numerals indicate the ROI number.
(TIF)

S2 Fig Change in 2D ^1^H-^13^C HSQC NMR peak intensities in 13 cultivars of rice straw following dilute acid pretreatment. The relative peak intensity of a component in the acid insoluble residue compared to the corresponding raw biomass is shown in each ROI (acid insoluble residue/raw biomass). ROIs with a <0.25 relative peak intensity are shown in blue, while ROIs with a >2.00 relative peak intensity are shown in red. Fold change increases or decreases are indicated by shades of red and blue according to the scale bar.
(TIF)

S1 Table NMR peak intensities showing highly positive (or negative) correlation coefficients with glucose, 5-HMF, and formate concentrations in liquid hydrolysate, and negative (or positive) correlations with xylose concentration in liquid hydrolysate and with the acid-insoluble residue yield. (DOCX)
