# Supplementary figures and images for: Changes in Lignin and Polysaccharide Components in 13 Cultivars of Rice Straw following Dilute Acid Pretreatment as Studied by Solution-State 2D 1H-13C NMR
Source: PLoS One. 2015 Jun 17;10(6):e0128417. doi: 10.1371/journal.pone.0128417 (PMC4470627; doi:10.1371/journal.pone.0128417)

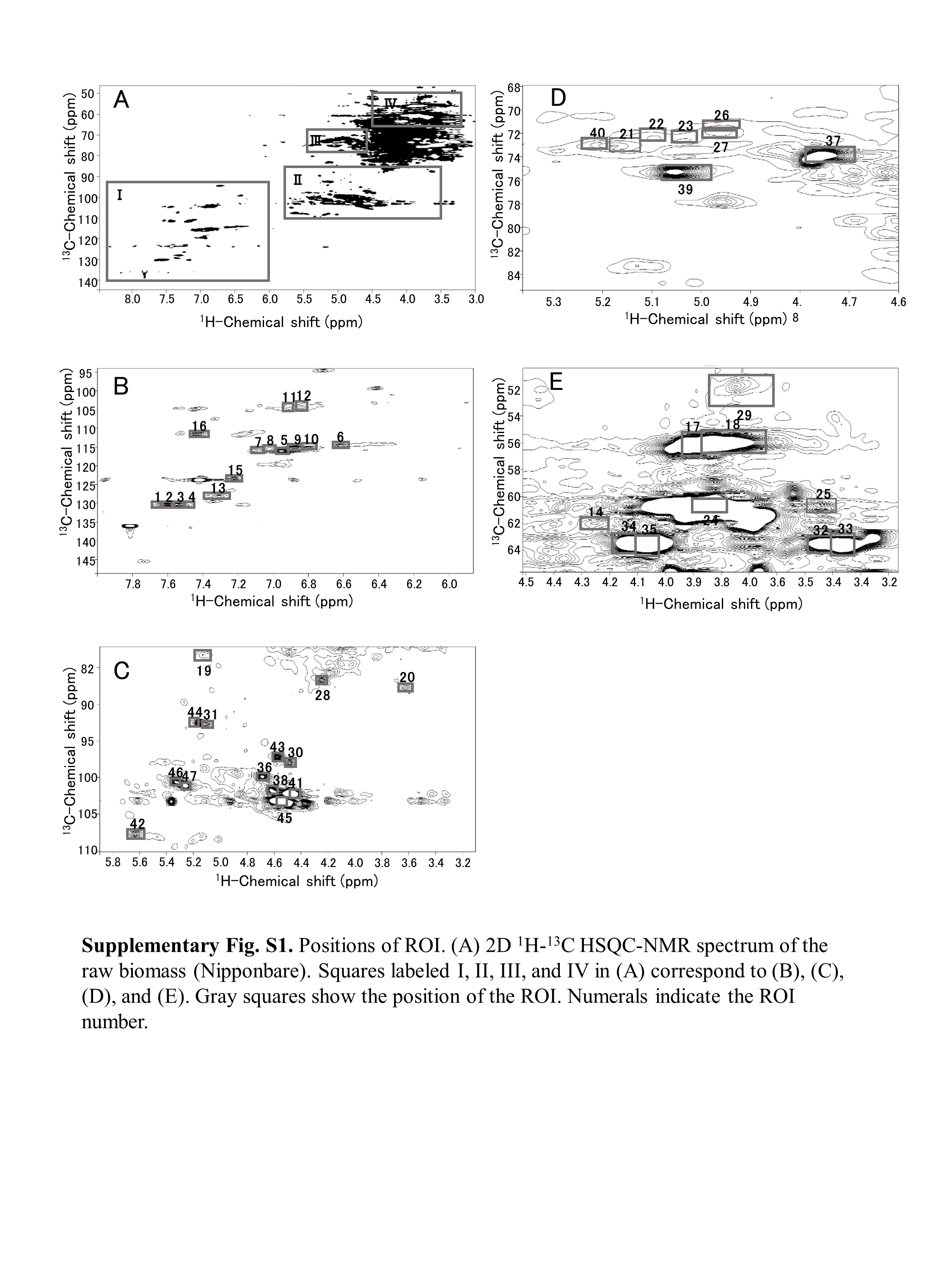

Supplement: S1 Fig — (A) 2D 1H-13C HSQC NMR spectrum of the raw biomass (Nipponbare). Squares I, II, III, and IV in (A) correspond to (B), (C), (D), and (E). Gray squares show the position of the ROI. Numerals indicate the ROI number. (TIF) [file pone.0128417.s001.tif]

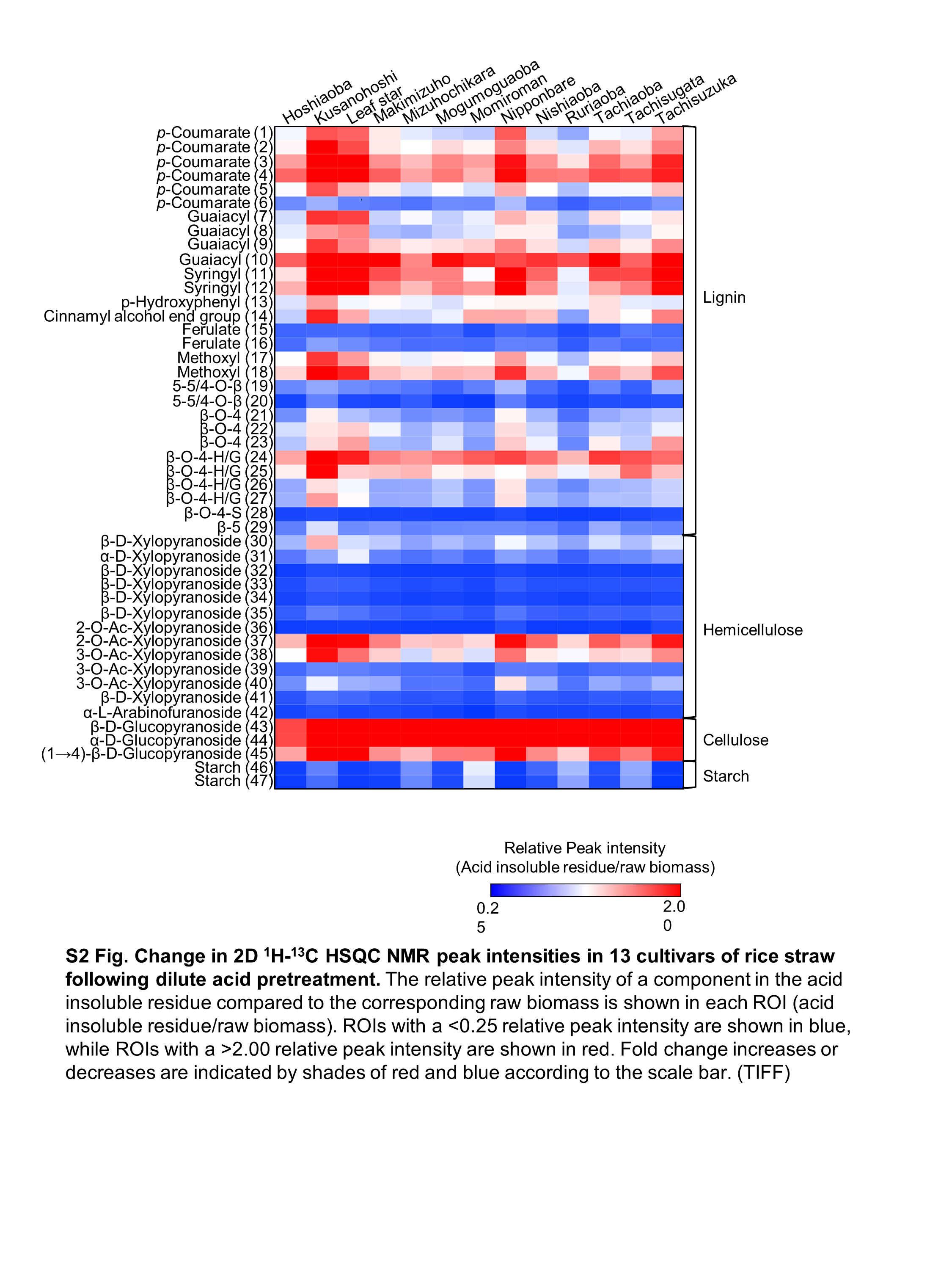

Supplement: S2 Fig — The relative peak intensity of a component in the acid insoluble residue compared to the corresponding raw biomass is shown in each ROI (acid insoluble residue/raw biomass). ROIs with a <0.25 relative peak intensity are shown in blue, while ROIs with a >2.00 relative peak intensity are shown in red. Fold change increases or decreases are indicated by shades of red and blue according to the scale bar. (TIF) [file pone.0128417.s002.tif]
