# Supplementary material for: Changes in Lignin and Polysaccharide Components in 13 Cultivars of Rice Straw following Dilute Acid Pretreatment as Studied by Solution-State 2D 1H-13C NMR
Source: PLoS One. 2015 Jun 17;10(6):e0128417. doi: 10.1371/journal.pone.0128417 (PMC4470627; doi:10.1371/journal.pone.0128417)
Supplement: S1 Table — (DOCX) [file pone.0128417.s003.docx]

**S1 Table.** **NMR peak intensities showing highly positive (or negative) correlation coefficients with glucose, 5-HMF, and formate concentrations in liquid hydrolysate, and negative (or positive) correlations with xylose concentration in liquid hydrolysate and with the weight of acid-insoluble residue.**

| ROI No. | Correlation coefficient (%) | | | | |
| --- | --- | --- | --- | --- | --- |
|  | Glucose | 5-HMF | Formate | Xylose | Acid-insoluble residue weight |
| ROI.47 | 0.94 | 0.83 | 0.71 | −0.95 | −0.95 |
| ROI.48 | 0.96 | 0.86 | 0.73 | −0.97 | −0.97 |
| ROI.33 | −0.79 | −0.69 | −0.45 | 0.80 | 0.72 |
| ROI.34 | −0.76 | −0.66 | −0.44 | 0.77 | 0.68 |
| ROI.35 | −0.80 | −0.69 | −0.48 | 0.80 | 0.73 |
| ROI.38 | −0.75 | −0.68 | −0.44 | 0.78 | 0.70 |
| ROI.39 | −0.81 | −0.74 | −0.50 | 0.82 | 0.74 |
| ROI.41 | −0.76 | −0.65 | −0.42 | 0.78 | 0.70 |
| ROI.42 | −0.75 | −0.67 | −0.45 | 0.77 | 0.68 |
